# Accurate and broadband manipulations of harmonic amplitudes and phases to reach 256 QAM millimeter-wave wireless communications by time-domain digital coding metasurface

**DOI:** 10.1093/nsr/nwab134

**Published:** 2021-07-29

**Authors:** Ming Zheng Chen, Wankai Tang, Jun Yan Dai, Jun Chen Ke, Lei Zhang, Cheng Zhang, Jin Yang, Lianlin Li, Qiang Cheng, Shi Jin, Tie Jun Cui

**Affiliations:** Institute of Electromagnetic Space, Southeast University, Nanjing 210096, China; State Key Laboratory of Millimeter Waves, Southeast University, Nanjing 210096, China; Center of Intelligent Metamaterials, Pazhou Laboratory, Guangzhou 510330, China; National Mobile Communications Research Laboratory, Southeast University, Nanjing 210096, China; Center of Intelligent Metamaterials, Pazhou Laboratory, Guangzhou 510330, China; Institute of Electromagnetic Space, Southeast University, Nanjing 210096, China; State Key Laboratory of Millimeter Waves, Southeast University, Nanjing 210096, China; State Key Laboratory of Terahertz and Millimeter Waves, City University of Hong Kong, Hong Kong, China; Institute of Electromagnetic Space, Southeast University, Nanjing 210096, China; State Key Laboratory of Millimeter Waves, Southeast University, Nanjing 210096, China; Institute of Electromagnetic Space, Southeast University, Nanjing 210096, China; State Key Laboratory of Millimeter Waves, Southeast University, Nanjing 210096, China; Center of Intelligent Metamaterials, Pazhou Laboratory, Guangzhou 510330, China; Institute of Electromagnetic Space, Southeast University, Nanjing 210096, China; Institute of Electromagnetic Space, Southeast University, Nanjing 210096, China; State Key Laboratory of Millimeter Waves, Southeast University, Nanjing 210096, China; Center of Intelligent Metamaterials, Pazhou Laboratory, Guangzhou 510330, China; State Key Laboratory of Advanced Optical Communication Systems and Networks, Department of Electronics, Peking University, Beijing 100871, China; Center of Intelligent Metamaterials, Pazhou Laboratory, Guangzhou 510330, China; Institute of Electromagnetic Space, Southeast University, Nanjing 210096, China; State Key Laboratory of Millimeter Waves, Southeast University, Nanjing 210096, China; Center of Intelligent Metamaterials, Pazhou Laboratory, Guangzhou 510330, China; Institute of Electromagnetic Space, Southeast University, Nanjing 210096, China; National Mobile Communications Research Laboratory, Southeast University, Nanjing 210096, China; Center of Intelligent Metamaterials, Pazhou Laboratory, Guangzhou 510330, China; Institute of Electromagnetic Space, Southeast University, Nanjing 210096, China; State Key Laboratory of Millimeter Waves, Southeast University, Nanjing 210096, China; Center of Intelligent Metamaterials, Pazhou Laboratory, Guangzhou 510330, China

**Keywords:** metasurface, time-domain digital coding, accurate and broadband harmonic control, 256 QAM millimeter-wave wireless communications

## Abstract

We propose a theoretical mechanism and new coding strategy to realize extremely accurate manipulations of nonlinear electromagnetic harmonics in ultrawide frequency band based on a time-domain digital coding metasurface (TDCM). Using the proposed mechanism and coding strategy, we design and fabricate a millimeter-wave (mmWave) TDCM, which is composed of reprogrammable meta-atoms embedded with positive-intrinsic-negative diodes. By controlling the duty ratios and time delays of the digital coding sequences loaded on a TDCM, experimental results show that both amplitudes and phases of different harmonics can be engineered at will simultaneously and precisely in broad frequency band from 22 to 33 GHz, even when the coding states are imperfect, which is in good agreement with theoretical calculations. Based on the fabricated high-performance TDCM, we further propose and experimentally realize a large-capacity mmWave wireless communication system, where 256 quadrature amplitude modulation, along with other schemes, is demonstrated. The new wireless communication system has a much simpler architecture than the currently used mmWave wireless systems, and hence can significantly reduce the hardware cost. We believe that the proposed method and system architecture can find vast application in future mmWave and terahertz-wave wireless communication and radar systems.

## Teaser

A new coding mechanism is proposed to realize extremely accurate and broadband manipulations of nonlinear harmonic distributions using a time-domain digital coding metasurface, reaching simplified-architecture 256 QAM (quadrature amplitude modulation) millimeter-wave wireless communications.

## INTRODUCTION

With the increasing demand for bandwidth-hungry mobile applications, such as cloud virtual reality (VR), holographic rendering and wireless medical services, the contradiction between huge capacity requirements and scarce spectrum resources has been markedly exacerbated. However, the commercial fourth-generation (4G) Long Term Evolution (LTE)-advanced and inchoate fifth-generation (5G) mobile communications struggle to satisfy this increasing demand. Against this background, the establishment of millimeter-wave (mmWave) wireless communication systems with larger bandwidths and potential Gb/s-level data rates is of utmost significance [1,2]. Nevertheless, compared with the current sub-6 GHz systems, larger bandwidths and higher carrier frequencies have induced substantial hardware constraints in mmWave implementations, including power amplifier nonlinearities and IQ imbalance [3,4]. In conventional wireless communication systems, the processes of signal modulating, digital-analog converting, mixing, filtering, amplifying and radiating are essential integration parts. Thus, the current large-scale antenna-array-based high-order mmWave transceivers based on traditional technologies are complicated and cost-prohibitive in many application scenarios [4]. Therefore, a cost-effective and energy-efficient architecture for mmWave communications is necessary to alleviate such problems [5].

Metamaterials and metasurfaces have attracted great interest in recent years for their versatile possibilities with regard to manipulating electromagnetic (EM) waves [6–12]. Moreover, embedded tunable devices, such as positive-intrinsic-negative (PIN) diodes and varactors, enable reconfiguration of the functions for the metasurface in real time, and hence provide new avenues to tailor EM waves dynamically [13–16]. Based on tunable and reconfigurable metasurfaces, the concept of a time-domain digital coding metasurface (TDCM) is proposed to digitally manipulate harmonics by changing external biasing with time [17,18]. Plenty of intriguing TDCM-based phenomena have been reported, such as harmonic beam steering [19–21], nonlinear polarization synthesis [22], Doppler cloaking [23,24] and broadband spectral camouflage [25]. Besides the advances at microwave frequencies, time-modulated optical metasurfaces have also aroused intense interest with regard to the development of new devices, such as non-reciprocal photonics and optical isolation [26–28].

In the field of wireless communications, TDCMs can find intriguing application by combining signal-processing algorithms in information science with real-time programmable elements. On one hand, TDCMs are exploited to smartly reconfigure the wireless propagation environments, thereby improving network coverages [29–33]; on the other hand, the ability of real-time manipulating responses (e.g. phase and amplitude) of EM waves empowers TDCMs to act as wireless transmitters without any requirements that have to be satisfied in conventional radio frequency (RF) chains [17,34–43]. In fact, the programmable meta-atoms of a TDCM actually serve as multiple antennas when it functions as a transmitter. As a result, transmit diversity techniques (e.g. Alamouti space-time coding) can easily be implemented in TDCMs to enhance the signal energy at the receivers and improve the performance of the whole system [44,45].

Though many advances have been reported in TDCM-boosted wireless communication, there are still two major drawbacks to be faced. The first one is the inherent tight dependence between the reflection amplitude and phase, giving rise to inevitable distortion of constellation diagrams in TDCM-based phase-shift keying (PSK) wireless systems [35–39]. Moreover, the dependence also hampers the realization of high-order modulation schemes, such as quadrature amplitude modulation (QAM), since these relatively complicated schemes need accurate and independent control of the amplitudes and phases.

Besides the above-mentioned limitations, the majority of the established TDCM-assisted wireless communication systems suffer from narrowband defects on account of the inherent dispersion of metasurfaces [17,34–43]. To overcome the problem of the dispersion nature of metasurfaces, broadband achromatic metalenses, with elaborate unit elements on their surfaces, are proposed [46–50]. However, such metalenses are invariably complicated and non-adjustable, hence it is difficult to use them in designing broadband TDCMs. As a result, ingenious strategies are urgently required to circumvent the above-mentioned constraints and fulfill accurate and broadband control of EM waves for next-generation wireless communication systems.

In this paper, we propose a cunning coding strategy to achieve exceedingly accurate and ultrabroadband control of harmonic waves based on TDCM technology. The reflection coefficient of a TDCM is periodically switched in time between two states whose intensities and phases are precisely and independently determined by the duty ratios and time delays of digital coding sequences. Thus, the required harmonic distributions can be generated at will. On the other hand, the broadband property of harmonic manipulations is realized via the ingenious coding strategy instead of complicated achromatic designs. Since only two different states are needed in our recipe, we embed PIN diodes in meta-atoms of the designed TDCM, which is then capable of operating in a broad frequency band (22–33 GHz) and validating the theory of harmonic control. Considering the challenging hardware constraints and expensive costs in current mmWave communication systems, the accurate and broadband harmonic-control paradigm has the potential to serve as a new technological route. To demonstrate this capability, we implement a 256 QAM scheme, which has 64 times the constellation points and four times the channel capacity of the quadrature phase-shift keying (QPSK) modulation. At the incidence of a monochromatic wave, the carrier wave is shifted to several harmonic waves under the control of digital coding sequences. Thus, we can directly synthesize the standardized constellations at the +1st-order harmonic via loading different coding sequences to the TDCM without using the complicated RF chain, exhibiting the relevant advantages of system simplicity, cost reduction and energy efficiency. Based on the proposed theory, encoding strategy and metasurface hardware, we realize a TDCM-boosted wireless communication system operating in the mmWave region from 27 to 31.15 GHz, in which 256 QAM and other modulation schemes are successfully accomplished. This study indicates the huge potential of the TDCM mechanism in future mmWave and terahertz-wave (THzWave) wireless communication systems with simple architecture, low cost and small energy consumption.

## ACCURATE CONTROL OF HARMONIC AMPLITUDES AND PHASES

**Figure 1.**
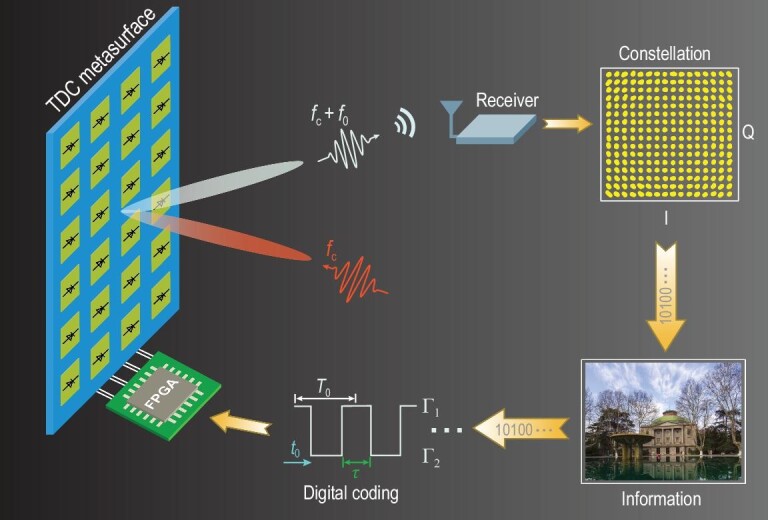
Conceptual illustration of the mmWave wireless communication system based on a TDCM. By applying control-voltage sequences with different duty ratios and time delays from the FPGA module to the PIN diodes, the incident monochromatic wave is converted to multiple harmonics, whose amplitudes and phases can be very accurately manipulated. The +1st-order harmonic is selected to realize the 256 QAM mmWave wireless communication system.

As depicted in Fig. 1, the proposed TDCM is composed of a two-dimensional array of reprogrammable elements embedded with PIN diodes. The reflection phase of the TDCM can be switched between two discrete states by using different control voltages loaded to the diodes. When the control-voltage sequence is time varying periodically with a time cycle ${T_0} = 1/{f_0}$, TDCM is competent for converting the incoming monochromatic plane wave with the frequency ${f_c}$ to multiple discrete harmonics. More specifically, the generated harmonics are regularly spread around the carrier frequency ${f_c}$ with a frequency interval ${f_0}$ (see Supplementary Note 1 for more details). Hence, we can tailor the spectra distributions to cater for diverse applications with ingeniously devised digital coding strategies.

We firstly define the two reflection coefficients of TDCM triggered by different control voltages as $| {{{\rm{\Gamma }}_1}} | \cdot {e^{j{\varphi _1}}}$ and${\rm{\ }}| {{{\rm{\Gamma }}_2}} | \cdot {e^{j{\varphi _2}}}$, where $| {{{\rm{\Gamma }}_1}} |$ and $| {{{\rm{\Gamma }}_2}} |$ as well as ${\varphi _1}$ and ${\varphi _2}$ are the reflection amplitudes and phases, respectively. Then the periodically varying control voltages in square waveforms generate a time-varying reflection coefficient${\rm{\ \Gamma }}( t )$. In our scenario, the elementary coding sequence is designed to be axisymmetric in a time period ${T_0} = {\rm{\ }}1/{f_0}$ with a duty ratio defined as $M\ = {\rm{\ }}\tau /{T_0}$, as illustrated in Fig. 1. By applying different duty ratios *M* and time delays *t*_0_ to the sequences, the amplitudes and phases of all harmonic waves could be manipulated independently. The coefficient of the *k*th-order harmonic is finally determined by

(1) \begin{equation*} {{{a_k} = \left\{ \begin{array}{@{}l@{}} {\rm{ }}M \cdot \left| {{\Gamma _1}} \right|{e^{j{\varphi _1}}} + (1 - M) \cdot \left| {{\Gamma _2}} \right|{e^{j{\varphi _2}}},\quad k = 0 \\ {r_0} \cdot M \cdot \left| {Sa(k\pi M)} \right|\\\, \cdot {e^{ - j\{ k{\omega _0}{t_0} + \frac{\pi }{2}[1 - {{( - 1)}^{\left\lfloor {\left| k \right| \cdot M} \right\rfloor }}]\} }},\quad k = \pm 1,{\rm{ }} \pm 2,{\rm{ }} \pm {\rm{3}} \cdot \cdot \cdot \end{array} \right.},} \end{equation*}

where ${r_0} = | {{{\rm{\Gamma }}_1}} |\ \cdot {e^{j{\varphi _1}}} - | {{{\rm{\Gamma }}_2}} | \cdot {e^{j{\varphi _2}}}$, $Sa(k\pi M$) denotes $\sin ( {k\pi M} )/k\pi M$, ${\omega _0} = \ 2\pi /{T_0}$ and ⌊ ⌋ indicates rounding down. Here, ${r_0}$ is determined by the two coding states of TDCM, revealing the combination of amplitude coefficients and initial phases that affect all harmonic amplitudes and phases simultaneously. As a result, with an apt selection of value ranges for *M* and *t*_0_, the amplitude and phase coefficients of the *k*th-order harmonic are respectively derived as

(2) \begin{equation*} \begin{array}{@{}l@{}} A \!=\! 2 \cdot \left| {{r_0}} \right| \cdot M \cdot Sa(k\pi M),\quad M \in \left[ {0,1/2\left| k \right|} \right],\\ \varphi \!=\! - k{\omega _0}{t_0} + {\varphi _0},\quad{t_0} \in \left[ {0,{T_0}/\left| k \right|} \right), \end{array} \end{equation*}

where $| {{r_0}} |$ and ${\varphi _0}$ denote the amplitude and phase of ${r_0}$, respectively. It is apparent that the two parameters are dispersive. Their impact on the harmonic control is discussed later. We herein assume that $| {{{\rm{\Gamma }}_1}} | = 1$, $| {{{\rm{\Gamma }}_2}} | = 1$, ${\varphi _1} = {0^\circ }$ and ${\varphi _2} = {180^\circ }$ for simplicity without losing generality. Then the harmonic amplitudes and phases are respectively determined by the duty ratios and time delays of the coding sequences (see Supplementary Note 2 for more details).

**Figure 2.**
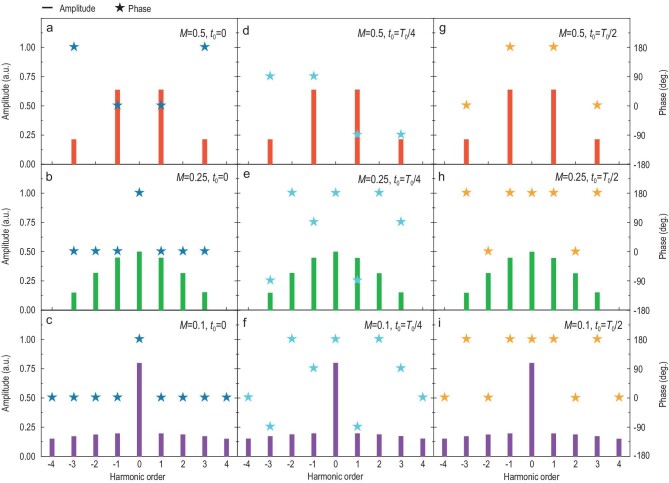
Calculated harmonic amplitudes and phases under different digital coding sequences, in which different duty ratios *M* and time delays *t*_0_ are taken into account. The same duty ratios $M\!=\! {\rm{\ }}0.5$ (a, d, g), $M\!=\! {\rm{\ }}0.25$ (b, e, h) and $M\!=\! {\rm{\ }}0.1$ (c, f, i) are adopted in each row, while the same time delays ${t_0}\! =\! {\rm{\ }}0$ (a, b, c), ${t_0}\! =\! {T_0}/4$ (d, e, f) and ${t_0}\! =\! {T_0}/2$ (g, h, i) are employed in each column, respectively.

To better illustrate the relationships between harmonic amplitude/phase and duty ratio/time delay, the calculated harmonic distributions of the reflected signals are presented under different coding schemes, as shown in Fig. 2. Here, the coding sequences in columns possess the changed duty ratios and constant time delay; while those in rows are opposite. As the duty ratio *M* is altered from 0.5 to 0.1, the amplitude distributions of the harmonics are changed accordingly, while the phase distributions remain unaltered or are reversed between the two phases differing by 180°. Likewise, when the time delays *t*_0_ are altered from 0 to *T*_0_/2, the phase distributions of the harmonics are changed accordingly, and the amplitude distributions keep unaltered. In short, by tailoring the duty ratios and time delays of the digital coding sequences, we are able to control the amplitudes and phases of the generated harmonic waves independently using TDCM without mutual influence.

## ROBUSTNESS OF THE CODING STRATEGY FOR BROADBAND OPERATIONS

In the above analysis, ideal TDCM states are presumed for simplicity. However, the actual states vary at different frequencies owing to the inherent dispersive property of TDCMs, which inevitably gives rise to narrow operating frequency bands in many TDCM-based applications. In contrast, our coding strategy possesses the robustness of broadband operation for certain scenarios, such as in wireless communications and harmonic beam steering, in which the relative relations between different amplitude and phase distributions really matter (see Supplementary Note 3 for more details).

**Figure 3.**
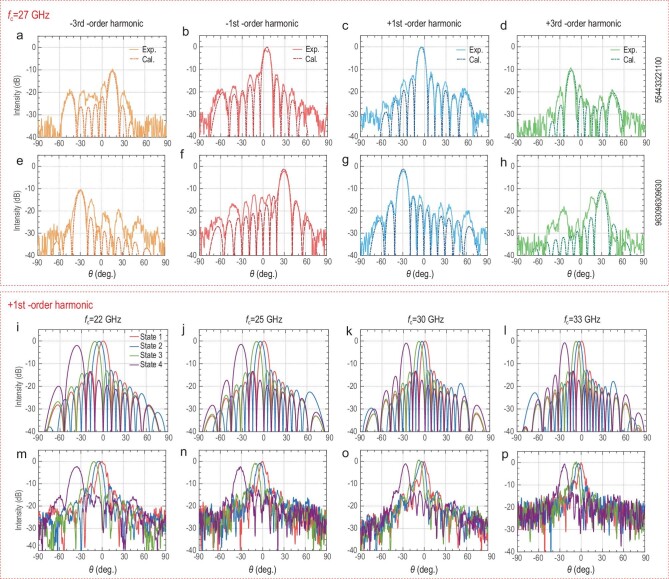
Accurate and broadband beam steering at different harmonics. (a–h) Calculated (dashed lines) and experimental (solid lines) directional diagrams for multi-order harmonics, including -3rd-order (orange lines), -1st-order (red lines), +1st-order (blue lines) and +3rd-order (green lines) harmonics, of the TDCM at *f*_c _= 27 GHz and *f*_0 _= 100 kHz under different phase distributions listed on the right. (i–l) Calculated and (m–p) experimental directional diagrams for the +1st-order harmonic of the TDCM at *f*_c _= 22, 25, 30, 33 GHz and *f*_0 _= 100 kHz under the phase distributions ‘000000000000’ (red lines), ‘554433221100’ (blue lines), ‘11-10-9876543210’ (green lines) and ‘963096309630’ (purple lines), respectively.

As depicted in Fig. 3, the harmonic beam steering experiments are carried out in a wide frequency

range (22–33 GHz) to verify the robustness and broadband property of the coding strategy. The theoretical calculations are based on the directional-diagram synthesis of a uniformly spaced array and the measurements are conducted in a microwave anechoic chamber. Detailed experimental setup is given in Supplementary Note 4. Here, every four columns of TDCM with 48 controllable columns share the same digital control signal and form a super-column. The width of the super-column is 5.6 mm, corresponding to 0.41λ at 22 GHz or 0.62λ at 33 GHz. To illustrate the accurate control of harmonic phases, we apply different time delays *t*_0_ to the coding sequences at each super-column to form harmonic phase distributions (see Supplementary Note 5 for the impact of duty ratios *M* on harmonic beam gains). Note that the duty ratios of all coding sequences are set as 0.5 for maximum harmonic conversion efficiency. The carrier wave frequency *f*_c_ and the modulating frequency *f*_0_ are set as 27 GHz and 100 kHz, respectively. In the experiment, 12 harmonic phase states are applied by adopting various time delays *t*_0_ to the coding sequences. The codes ‘0’, ‘1’, ‘2’, ‘3’, ‘4’, ‘5’, ‘6’, ‘7’, ‘8’, ‘9’, ‘10’ and ‘11’ are used to represent the phase states when *t*_0_ equals 0, $\frac{{{T_0}}}{{12}}$, $\frac{{{T_0}}}{6}$, $\frac{{{T_0}}}{4}$, $\frac{{{T_0}}}{3}$, $\frac{{5{T_0}}}{{12}}$, $\frac{{{T_0}}}{2}$, $\frac{{7{T_0}}}{{12}}$, $\frac{{2{T_0}}}{3}$, $\frac{{3{T_0}}}{4}$, $\frac{{5{T_0}}}{6}$ and $\frac{{11{T_0}}}{{12}}$. According to Equation (1), these codes can bring about different phase states to different harmonics. Specifically, {0, $\frac{\pi }{6}$, $\frac{\pi }{3}$, $\frac{\pi }{2}$, $\frac{{2\pi }}{3}$, $\frac{{5\pi }}{6}$, ${\rm{\pi }}$, $\frac{{7\pi }}{6}$, $\frac{{4\pi }}{3}$, $\frac{{3\pi }}{2}$, $\frac{{5\pi }}{3}$, $\frac{{11\pi }}{6}$} for the +1st-order harmonic, {0, $\frac{{11\pi }}{6}$, $\frac{{5\pi }}{3}$, $\frac{{3\pi }}{2}$, $\frac{{4\pi }}{3}$, $\frac{{7\pi }}{6}$, ${\rm{\pi }}$, $\frac{{5\pi }}{6}$, $\frac{{2\pi }}{3}$, $\frac{\pi }{2}$, $\frac{\pi }{3}$, $\frac{\pi }{6}$} for the -1st-order harmonic, {0, $\frac{\pi }{2}$, ${\rm{\pi }}$, $\frac{{3\pi }}{2}$, $0$, $\frac{\pi }{2}$, ${\rm{\pi }}$, $\frac{{3\pi }}{2}$, $0$, $\frac{\pi }{2}$, ${\rm{\pi }}$, $\frac{{3\pi }}{2}$} for the +3rd-order harmonic and {0, $\frac{{3\pi }}{2}$, ${\rm{\pi }}$, $\frac{\pi }{2}$, $0$, $\frac{{3\pi }}{2}$, ${\rm{\pi }}$, $\frac{\pi }{2}$, $0$, $\frac{{3\pi }}{2}$, ${\rm{\pi }}$, $\frac{\pi }{2}$} for the -3rd-order harmonic.

As shown in Fig. 3a–h, two phase distributions ‘554433221100’ and ‘963096309630’ are applied to the TDCM, and the solid and dashed lines represent the experimental and calculated results, respectively. Meanwhile, the curves of different harmonics are distinguished via the colors of orange, red, blue and green. Owing to the accurate manipulations of harmonic phases, the main lobes of measured directional diagrams have excellent agreements with the calculations. The gains of the 3rd-order harmonics are ∼9.5 dB lower than those of the 1st-order harmonics, demonstrating their amplitude relationship. However, the side lobes of experimental patterns are relatively higher, as the low side lobes are inundated by environmental noises. Nevertheless, the current measurements strongly prove the accuracy of our coding strategy over the harmonic phase control. Then we further test the diverse directional diagrams at the +1st-order harmonic of the carrier waves *f*_c _= 22, 25, 30 and 33 GHz, as shown in Fig. 3i–p. The modulating frequency *f*_0_ is set as 100 kHz. The states 1, 2, 3 and 4 correspond to the phase distribution ‘000000000000’, ‘554433221100’, ‘11-10-9876543210’ and ‘963096309630’, respectively. When the phase distribution varies from 1 to 4, the main lobes are gradually steered to wider angles. The directional diagrams under the same phase distributions are different under diverse operating frequencies. This is because the harmonic phase distributions are achromatic and merely determined by time delays *t*_0_ of the coding sequences, while the relative width of the super-column is dispersive. By comparing Fig. 3i–l with Fig. 3m–p, the experimental results align well with the calculations at the +1st-order harmonic of the carrier waves from 22 to 33 GHz (see Supplementary Note 6 for detailed deflection angles). So, 40% relative bandwidth of the broadband harmonic beam steering is achieved with the TDCM under our coding strategy.

**Figure 4.**
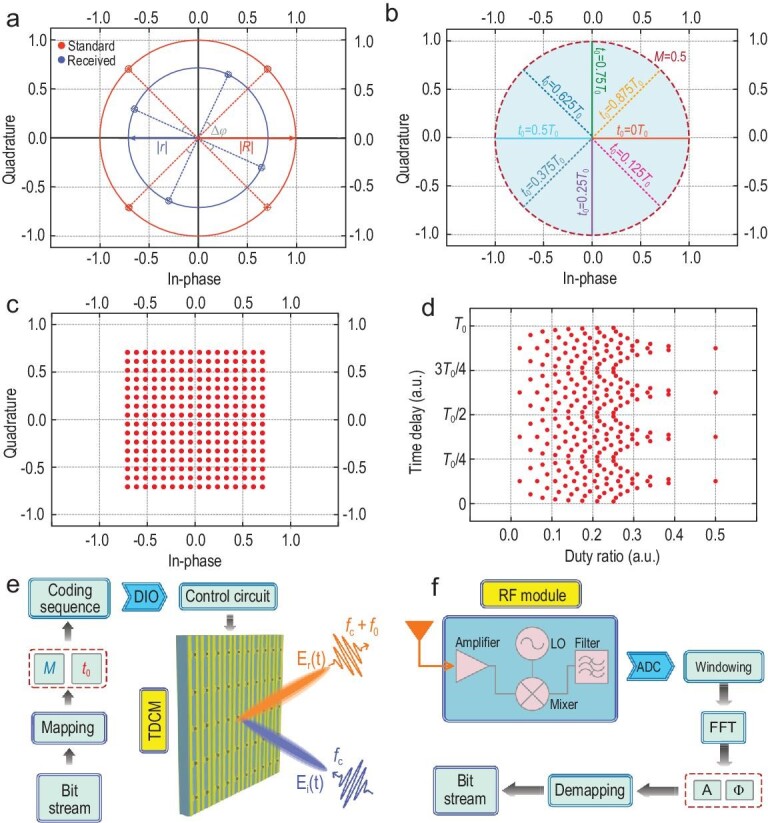
Constellation diagrams and block diagram of the proposed mmWave wireless communication system. (a) Received and standard constellation diagrams of the QPSK modulation. (b) In-phase and quadrature components of ${\rm{\Upsilon }}_A^\varphi $ with different combinations of *M* (ranging from 0 to 0.5) and *t*_0_ (0, 0.125*T*_0_, 0.25*T*_0_, 0.375*T*_0_, 0.5*T*_0_, 0.625*T*_0_, 0.75*T*_0_ and 0.875*T*_0_). (c) The constellation diagrams of the 256 QAM scheme. (d) The scatter diagram of the 256 QAM scheme in the duty ratio and time delay coordinate system. (e) The transmitting terminal, in which the transmitted bitstreams are mapped into the coding sequences with the corresponding duty ratios and time delays and then loaded to the TDCM via the Digital Input and Output (DIO) module and control circuit. (f) The receiving terminal, in which the received signal at ${f_c} + {f_0}$ is firstly handled by the RF module, then goes through a series of baseband signal processing procedures and finally is de-mapped to bitstreams.

Similar to the beam steering scenario, in the case of wireless communications, at a given carrier frequency, the difference of two coding states attaches an extra amplitude coefficient $| {{r_0}} |$ and phase coefficient ${\varphi _0}$ to the respective amplitude and phase distributions of all harmonics, as seen in Equation (2). As a result, the distortion upon the harmonic manipulation caused by ${r_0}$ could be corrected via normalization. For instance, Fig. 4a illustrates the received signal and standard diagram for the QPSK modulation, while the constellation set for a standard QPSK modulation is {${e^{j\frac{\pi }{4}}}$, ${e^{j\frac{{3\pi }}{4}}}$, ${e^{j\frac{{5\pi }}{4}}}$, ${e^{j\frac{{7\pi }}{4}}}$}. To achieve the maximum energy efficiency for the modulation, the +1st-order harmonic is selected. Here, the duty ratio *M* is set at 50%, while the time delay *t*_0_ is $\frac{{7{T_0}}}{8}$, $\frac{{5{T_0}}}{8}$, $\frac{{3{T_0}}}{8}$ and $\frac{{1{T_0}}}{8}$, respectively. Furthermore, given the distortion caused by TDCM and the amplitude attenuation $| {{r_f}} |$ as well as the additional phase variation ${\varphi _f}$ during free-space transmission, the final received signal set is $\frac{2}{\pi } \cdot | {{r_0}} | \cdot | {{r_f}} | \cdot {e^{j( {{\varphi _0} + {\varphi _f}} )}} \cdot \{ {{e^{j\frac{\pi }{4}}},{\rm{\ }}{e^{j\frac{{3\pi }}{4}}},{\rm{\ }}{e^{j\frac{{5\pi }}{4}}},{\rm{\ }}{e^{j\frac{{7\pi }}{4}}}} \}$. Thus, we could transfer the received signal to the standard constellation set via a simple normalization. It is worth noting that although the QPSK modulation is taken as an example, the method of normalization is compatible with other modulation schemes.

From the above analyses, we note that the imperfect coding states of TDCM have no impact on the relative relations of harmonic amplitudes and phases under distinct coding sequences. This important feature strongly facilitates the broadband operations of harmonic beam steering and wireless communications.

## HIGH-ORDER MODULATIONS FOR MMWAVE WIRELESS COMMUNICATIONS

Despite the tremendous success of traditional-hardware frameworks in sub-6 GHz wireless communication systems, the higher operating frequency band and broader signal bandwidth in the mmWave region pose a great challenge to the conventional technical route. As a result, it is highly valuable to explore the feasibility of implementing simplified-architecture mmWave communication systems. The proposed digital coding strategy makes this idea a reality due to its accurate and broadband harmonic manipulations. For experimental demonstration, up to 256 QAM schemes are realized.

Without loss of generality, we assume that $| {{{\rm{\Gamma }}_1}} | = 1$, $| {{{\rm{\Gamma }}_2}} | = 1$, ${\varphi _1} = {0^\circ }$ and ${\varphi _2} = {180^\circ }$ for simplicity. As discussed above, the combination of $M \in [ {0,\ 1/2| k |} ]$ and ${t_0} \in [ {0,{\rm{\ }}{T_0}/| k |} )$ is eligible for obtaining precise manipulations of the *k*th-order harmonic amplitude ${A^{{k^{th}}}} \in [ {0,{\rm{\ }}2/| k |\pi } ]$ and harmonic phase ${{\rm{\Phi }}^{{k^{th}}}} \in [ {0,{\rm{\ }}2\pi } )$. Thus, it is possible for any *k*th-order harmonic to fulfill the intricate signal modulation. As presented in Fig. 2, the 1st-order harmonics have much higher converting efficiency than other harmonics, hence a +1st-order harmonic is chosen to illustrate the modulating procedure via the 1-bit TDCM (see Supplementary Note 7 for more details).

We herein define ${\rm{\Upsilon }}_A^\varphi = \ A{e^{j\varphi }}$ as an equivalent harmonic reflection coefficient. Then the pivotal issue lies in establishing correlations between the equivalent harmonic reflection coefficients and the message symbols. Actually, the time-varying ${\rm{\Upsilon }}( t )$ can be decomposed into the supposition of a series of individual equivalent harmonic reflection coefficients, thus

(3) \begin{equation*} {\rm{\Upsilon \ }}\!( t) = {{\rm{\Upsilon }}_s}\ \cdot g\!\left( t \right),\quad 0 \le t \le {T_0},\ {{\rm{\Upsilon }}_s} \in S, \end{equation*}

where ${{\rm{\Upsilon }}_s}$ represents the chosen message symbol belonging to a set of constellation points *S* with the cardinal number ${|}$*S*${|}$, and $g( t )$ is a basic pulse function. In wireless communication systems, each message symbol${\rm{\ }}{{\rm{\Upsilon }}_s}$ represents log_2_${|}$*S*${|}$-bits data.

As an illustrative example, a set of constellation points *S* are defined as $\{ {{\rm{\Upsilon }}_1^{0^\circ },\ {\rm{\Upsilon }}_1^{180^\circ }} \}$ for a binary phase shift keying (BPSK) modulation. Each ${\rm{\Upsilon }}_A^\varphi $ could be mapped to a coding sequence with a combination of the duty ratio *M* and time delay *t*_0_ via Equation (2), where the harmonic amplitudes and phases are normalized. For convenience, ${\rm{\Lambda }}_M^{{t_0}}$ represents the combination of the duty ratio and time delay applied to the coding sequence (e.g. ${\rm{\Lambda }}_{0.5}^{{T_0}/2}$ represents the coding sequence with duty ratio *M *= 0.5 and time delay *t*_0 _= *T*_0_/2). Figure 4b depicts the position of ${{\rm{\Upsilon }}_s}$ in the In-phase/Quadrature (I/Q) plane when *M* ranges from 0 to 0.5 and *t*_0_ ranges from 0 to *T*_0_. It is clearly observed that any required constellation diagrams can be synthesized with an apt selection of ${\rm{\Lambda }}_M^{{t_0}}$. Hence, the set of constellation points *S* for the BPSK modulation could be redefined as $\{ {{\rm{\Lambda }}_{0.5}^{0{T_0}},\ {\rm{\Lambda }}_{0.5}^{{T_0}/2}} \}$ when mapped to the actual coding sequences.

To better illustrate the process of mapping the constellation diagrams to actual coding sequences, we establish a duty ratio and time delay (*M*-*t*_0_) coordinate system. Then we could obtain the coding sequences of 256 QAM based on the scatter diagrams in the *M*-*t*_0_ coordinate system. Figure 4c illustrates the square constellation diagram of 256 QAM, showing that the constellation points are centrosymmetric to the coordinate origin and axisymmetric to the I/Q axes. These symmetrical properties elicit delicate regularity on the scatter points in the *M*/*t*_0_ plane too. For instance, the points are reduplicative in each adjacent quadrant when their azimuth angles are rotated by 90° in the I/Q plane. Consequently, the points in the *M*/*t*_0_ plane are also duplicated when the time delay *t*_0_ varies by 0.25*T*_0_, as vividly depicted in Fig. 4d. Therefore, the constellation points sharing the same distance to the origin are distributed in the line parallel to the *t*_0_-axis, whereas those sharing the same azimuth angle are distributed in the line parallel to the *M*-axis. Then the wireless communication process can be eventually implemented based on the performed mapping. Please refer to Supplementary Note 8 for the detailed mapping relationship.

Figure 4e and f presents the block diagram of the proposed mmWave communication system that is produced in reality (see the Experimental Verification part). As anticipated, due to the precise control of ${\rm{\Upsilon }}_A^\varphi $ and the robustness of the coding strategy, we successfully realize 256 QAM in the + 1^st^-order harmonic along with other modulation schemes. To better illustrate the whole communication process, we still take the BPSK modulation as an example. At the transmitting terminal (Fig. 4e), the baseband module generates bitstreams (such as 10100…) of the transmitted information (e.g. videos or pictures). Then all bitstreams are mapped to the corresponding coding sequences of TDCM, where ‘0’ is mapped to ${\rm{\Lambda }}_{0.5}^{0{T_0}}$ and ‘1’ is mapped to ${\rm{\Lambda }}_{0.5}^{{T_0}/2}$. Subsequently, the Field Programmable Gate Array (FPGA) module controls the Digital Input and Output (DIO) module to produce a series of digital signals to be loaded to TDCM through a control circuit. Finally, EM waves carrying the digital information at the + 1^st^-order harmonic are transmitted.

Figure 4f shows the block diagram of the receiver. Here, the received EM waves are processed through the RF module, in which the down-converting frequency is set to ${f_c} + {f_0}$. Then the recovered baseband signals through the Analog to Digital Converter (ADC) module are transformed to the frequency domain using the fast Fourier transform (FFT). Finally, by detecting *A* and${\rm{\ }}\varphi $ of each message symbol, where ${\rm{\Upsilon }}_1^{0^\circ }$ is de-mapped to ‘0’ and ${\rm{\Upsilon }}_1^{180^\circ }$ is de-mapped to ‘1’, the corresponding transmitted information is recovered. It is worth pointing out that, although the above-mentioned design process is for the BPSK modulation, it is apt for other modulations like 256 QAM with only a slight difference in the bitstream mapping and de-mapping steps.

We remark that the realization of accurate control over harmonic amplitudes and phases provides a degree of freedom compared with the previous phase-only metasurfaces. Hence, we significantly facilitate the functioning of metasurfaces in high-order modulation scenarios in a low-cost and high-efficiency manner, which may pave the way for better mmWave and THzWave wireless communications in the future.

## EXPERIMENTAL VERIFICATION

**Figure 5.**
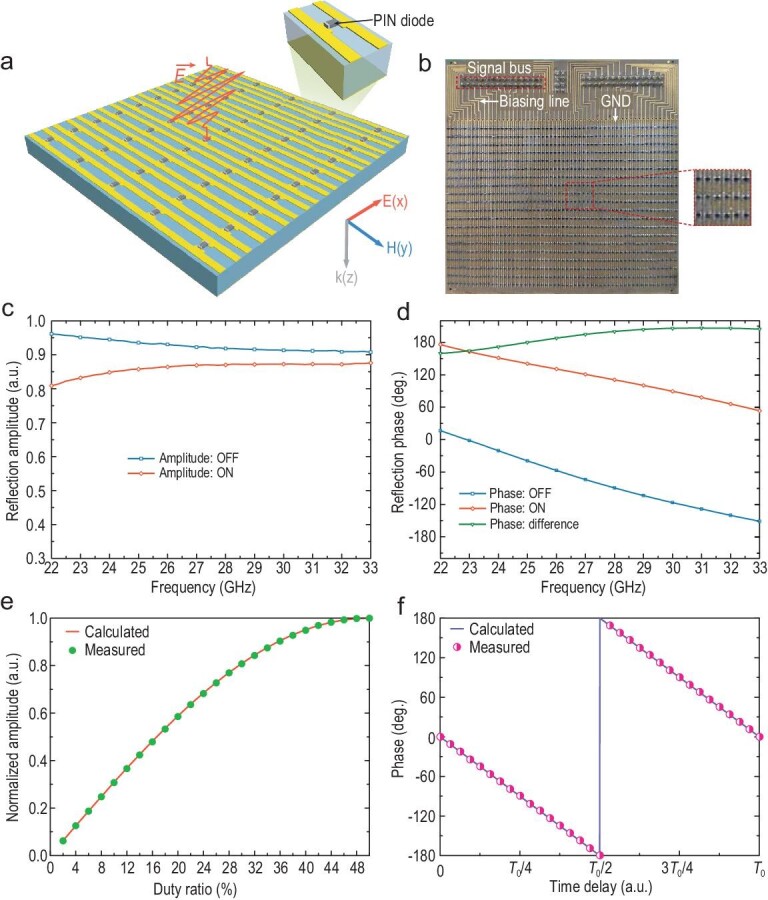
Design of the TDCM and measured results. (a) Schematic view of the designed 1-bit TDCM and zoomed view of the coding element loaded with a PIN diode. (b) Fabricated archetype of the TDCM and magnified view of the meta-atoms. (c and d) Numerically computed reflection amplitude and phase responses of the coding element under different PIN diode states. (e) Measured (dotted line) and calculated (solid line) amplitudes of the +1st-order harmonic versus the duty ratio at *f*_c _= 27 GHz and *f*_0 _= 125 kHz. (f) Measured (dotted line) and calculated (solid line) phases of the +1st-order harmonic versus the time delay at *f*_c _= 27 GHz and *f*_0 _= 125 kHz.

To validate the proposed harmonic-manipulation theory and realize the mmWave wireless communication system, we design a broadband 1-bit TDCM, as shown in Fig. 5a, where the 3D schematic view of the meta-element is especially enlarged. Each element consists of two rectangular patches printed on substrate (Rogers RT5880LZ) with the dielectric constant 1.96, loss tangent 0.002 and thickness 1.52 mm. A PIN diode (M/A-COM MADP-000907-14020x) is loaded between the two patches, and its forward voltage determines the coding states of the TDCM. The element size is 1.4 × 2.8 mm^2^, which corresponds to 0.126 × 0.252 ${\rm{\lambda }}$^2^ at a frequency of 27 GHz. The element geometries are optimized to obtain a 180° reflection-phase difference when the PIN diode is switched between ON and OFF states (see Supplementary Note 9 for more details). As presented in Fig. 5b, we fabricate a TDCM sample composed of $56 \times 20$ elements, whose columns are connected together and share a common control voltage via the bias lines (48 of the columns are actually used due to the constraints of DIO lines). Meanwhile, all ground (GND) lines are connected to the metallic backboard via through holes. Finally, the signal and GND lines are integrated to an interface, which links the DIO lines.

Full-wave and circuit joint simulations are conducted using commercial software, CST Microwave Studio 2016, in which the unit-cell boundary conditions are applied to the *x* and *y* directions, and two Floquet ports are used along the ±*z* directions in the simulation set-up. Under the illumination of plane waves with *x*-polarized electric fields, the simulated reflection amplitude and phase responses are plotted with varied forward voltages (0 or 1.42 V), as shown in Fig. 5c and d, respectively. We observe that the reflection-phase difference is ∼180° and the reflection amplitudes are larger than 0.8 from 22 to 33 GHz. It is worth noting that the broadband and accurate harmonic control in the mmWave region have been realized using the designed TDCM and the proposed coding algorithm.

To verify the effectiveness of the harmonic control, a series of time-varying coding sequences with different duty ratios *M* and time delays *t*_0_ are loaded to TDCM under the illumination of EM waves with the frequency *f*_c _= 27, 29 and 31.15 GHz. As a result, despite a slight deviation, the measurements strongly support the proposed broadband theory on harmonic manipulations. Please refer to Supplementary Note 10 for detailed results and discussions. Since the +1st-order harmonic is specially selected to realize the wireless communication system, its normalized amplitude curve versus the duty ratio, and phase curve versus the time delay are especially measured, as shown in Fig. 5e and f. Owing to the robustness of our method, the measured results are in excellent agreement with theoretical calculations, and thus the complicated high-order modulations in wireless communications are achievable.

**Figure 6.**
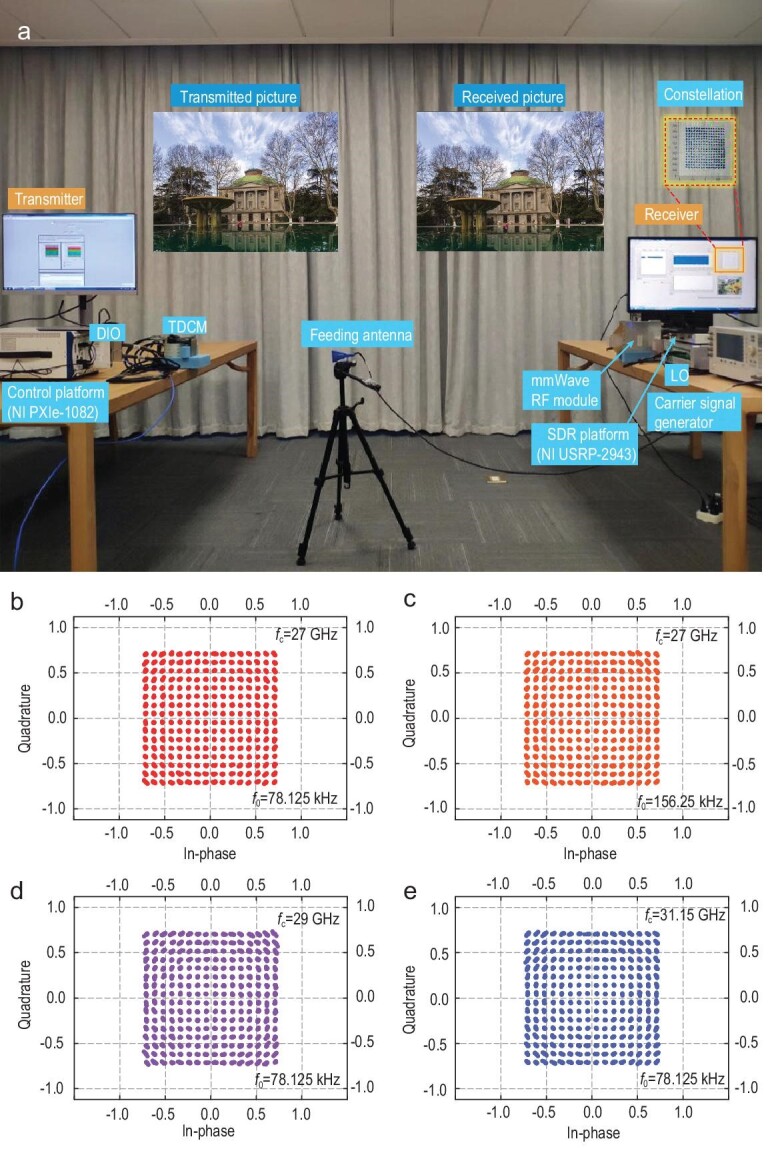
Photograph of the experimental set-up and measured constellation diagrams of the 256 QAM scheme with different carrier and modulation frequencies. (a) Photograph of the mmWave wireless communication system based on the 1-bit TDCM, in which 256 QAM is conducted along with other modulation schemes through a platform in the mmWave region. (b and c) The 256 QAM schemes with 78.125 kHz and 156.25 kHz frequency intervals at *f*_c _= 27 GHz, respectively. (d and e) The 256 QAM schemes with a frequency interval of 78.125 kHz at *f*_c _= 29 GHz and 31.15 GHz, respectively.

Based on the fabricated TDCM, we build an mmWave wireless communication system at 27–31.15 GHz by using the proposed coding strategy, as depicted in Fig. 6a. During experiments, a microwave signal generator (Keysight E8267D) connected with a linearly polarized horn antenna serves as the excitation source to supply the desired carrier waves at fixed frequencies. Meanwhile, a control platform (NI PXIe-1082) integrating a high-speed bus controller, an FPGA module (NI PXIe-7966R), a synchronous clock module (NI PXIe-6674T) and a DIO module (NI 6581B) are used to encode the desired coding sequences, which are eventually loaded to the TDCM through DIO lines. Since the system operates in the mmWave region, which has a frequency much higher than that of the general software-defined receiver (SDR), a heterodyne architecture is used at the receiving terminal. More specifically, the received signals are first down-converted to a sub-6 GHz frequency band using an RF module, of which the local oscillator (LO) frequency and operating frequency band are 25.2 GHz and 27–29 GHz, respectively. Moreover, another RF module working at 31.15 GHz with an intermediate frequency (IF) of 2.55 GHz is also employed in the experiment. Then an SDR platform (NI USRP-2943) is configured to demodulate the converted signals, including the down-conversion, sampling and baseband operation. Finally, the transmitted bitstreams are recovered and the transmitted information (the pictures of the auditorium and library of Southeast University) as well as the real-time constellation diagrams are displayed on the screen. As presented in Fig. 6a, a picture from the transmitter can be accurately recovered at the receiving end, which validates the effectiveness of the proposed communication system (see Supplementary Video for more details).

Using the above-depicted hardware platform, we successfully implement 256 QAM at different operating frequencies *f*_c_ and different harmonic intervals *f*_0_. The correspondingly measured constellation diagrams are shown in Fig. 6b–e. Please refer to Supplementary Note 11 for other modulation schemes. In communications, the constellation is a straightforward representation of the relation between the baseband signals, where the signals are depicted in a complex I/Q plane. As expected, the measured constellations agree extremely well with the standard constellations. Figure 6b and c presents the measured constellations at *f*_c _= 27 GHz with different frequency intervals (*f*_0 _= 78.125 and 156.25 kHz), respectively. Overall, the results indicate that high-precision and high-order modulations can be realized using the proposed coding strategy. Furthermore, the received constellations at different carrier frequencies (*f*_c _= 29 and 31.15 GHz) with the frequency interval *f*_0 _= 78.125 kHz are illustrated in Fig. 6d and e, demonstrating the broadband property of the harmonic-control mechanisms. Since the duty ratios *M* are set as <50% during the process of data transmission, on average less than half of all 960 (48 × 20) PIN diodes are switched on, each of which has 1.42 V and 1 mA forward voltage and current on average. As a result, the power consumption of the TDCM is <1 W. Finally, we can envision the potential application of our TDCM and modulation schemes in the mmWave and THzWave regions in future 6G wireless communication systems.

## CONCLUSION

We proposed an ingenious digital coding strategy to control harmonic amplitudes and phases simultaneously with very high accuracy by elaborately designing the duty ratios and time delays of the digital coding sequences. The robustness of the coding strategy also meant that the proposed TDCM operated in a broad frequency band. Based on precise manipulations of harmonics and broadband features, we set up a novel mmWave wireless communication system with the +1st-order harmonic acting as the carrier waves, which successfully realized the 256 QAM modulations. Compared with conventional wireless communication architectures in the mmWave band that require complicated baseband operations and expensive RF devices, our simplified wireless system circumvented many difficulties and reached a very high level of performance at low cost. We believe that the proposed coding strategy, wave manipulation mechanism and system architecture can be applied in many future 6G communications and new radar systems.

## Supplementary Material

nwab134_Supplemental_Files
