# Supplementary material for: Accurate and broadband manipulations of harmonic amplitudes and phases to reach 256 QAM millimeter-wave wireless communications by time-domain digital coding metasurface
Source: Natl Sci Rev. 2021 Jul 29;9(1):nwab134. doi: 10.1093/nsr/nwab134 (PMC8783670; doi:10.1093/nsr/nwab134)
Supplement: nwab134_Supplemental_Files [file nwab134_supplemental_files.zip › Supplementary_Data.docx]

**Supplementary Information for**

**Accurate and broadband manipulations of harmonic amplitudes and phases to reach 256QAM millimeter-wave wireless communications by time-domain digital coding metasurface**

Ming Zheng Chen1,2,6,†, Wankai Tang3,6,†, Jun Yan Dai1,2,4,†, Jun Chen Ke1,2, Lei Zhang1,2,6, Cheng Zhang1, Jin Yang1,2,6, Lianlin Li5,6, Qiang Cheng1,2,6,*, Shi Jin1,3,6,*, and Tie Jun Cui1,2,6,*

**1** *Institute of Electromagnetic Space, Southeast University, Nanjing 210096, China*

**2** *State Key Laboratory of Millimeter Waves, Southeast University, Nanjing 210096, China*

**3** *National Mobile Communications Research Laboratory, Southeast University, Nanjing 210096, China*

**4***State Key Laboratory of Terahertz and Millimeter Waves, City University of Hong Kong, Hong Kong SAR 999077, China*

**5** *State Key Laboratory of Advanced Optical Communication Systems and Networks, Department of Electronics, Peking University, Beijing 100871, China*

**6***Pazhou Laboratory, Guangzhou 510330, China*

† These authors contribute equally to this work.

* Corresponding authors: [qiangcheng@seu.edu.cn](mailto:qiangcheng@seu.edu.cn); [jinshi@seu.edu.cn](mailto:jinshi@seu.edu.cn); [tjcui@seu.edu.cn](mailto:tjcui@seu.edu.cn)

**This Supplementary Information includes:**

Supplementary Notes 1 to 11

Supplementary Figures S1 to S9

Supplementary Tables S1 to S9

Supplementary Equations S1 to S8

Supplementary Video 1

Supplementary References

**Supplementary Note 1: Principle of harmonic generation via TDCM**

We start by considering a reflective TDCM illustrated by a plane wave. The EM wave could be modulated by TDCM with a time-varying reflection coefficient , and the reflected EM wave could be written as

, (S1)

where and denote the reflected and incident electric fields, respectively [1]. Under the excitation of a monochromatic signal at frequency *f*c, the reflected field in the frequency domain is derived as

. (S2)

When the reflection coefficient is controlled by a periodically time-varying control voltage, it could be mathematically rewritten as a sum of series [1], where . Hence the reflected field is obtained as

, (S3)

where is the angular frequency settled by the period *T*0 of the coding sequence, and is the complex Fourier series coefficient at the frequency of . With the aid of elaborately designed periodic coding sequences, we could synthesize arbitrarily spectra distributions according to Eq. (S3) [1].

**Supplementary Note 2: Accurate controls of harmonic amplitudes and phases via duty ratios and time delays of the digital coding sequences**

As discussed in the main text, the elementary coding sequence is axisymmetric within a time period. Thus the time-varying reflection coefficient reads

, (S4)

in which *m* is the natural number. Then the complex Fourier series coefficients in Eq. (S3) are calculated through the Fourier transform as

, (S5)

in which denotes . The calculated harmonic amplitude (solid line) and phase (dashed line) curves versus the duty ratio are shown in Fig. S1, in which ||=1, ||=1, =0°, and =180°. Under the coding strategy characterized by Eq. (S4), the amplitudes of the ±1st-order harmonics are symmetrical with respect to , and monotonically increase when with the peak value at . The amplitudes of higher-order harmonics monotonically increase when with the peak value at and repeat the waveform of when . It is worth noting that when the amplitudes of the ±1st-order harmonics are altered with different duty ratios of the coding sequences, their phases are identical with zero, as depicted in Fig. S1a (dashed line). The phenomena of phase invariance are similar for higher-order harmonics except that the phases of them reverse between 0° and 180° in each interval of when *M* increases from 0 to 1, as illustrated in Fig. S1b and c (dashed lines).

Hence we can control the *k*th-order harmonic amplitudes via tailoring the duty ratios *M* of the digital coding sequences characterized by Eq. (S4). More importantly, when *M* is restricted to , we can obtain a one-to-one correspondence between the *k*th-order harmonic amplitude and the duty ratio as well as the invariable harmonic phase.


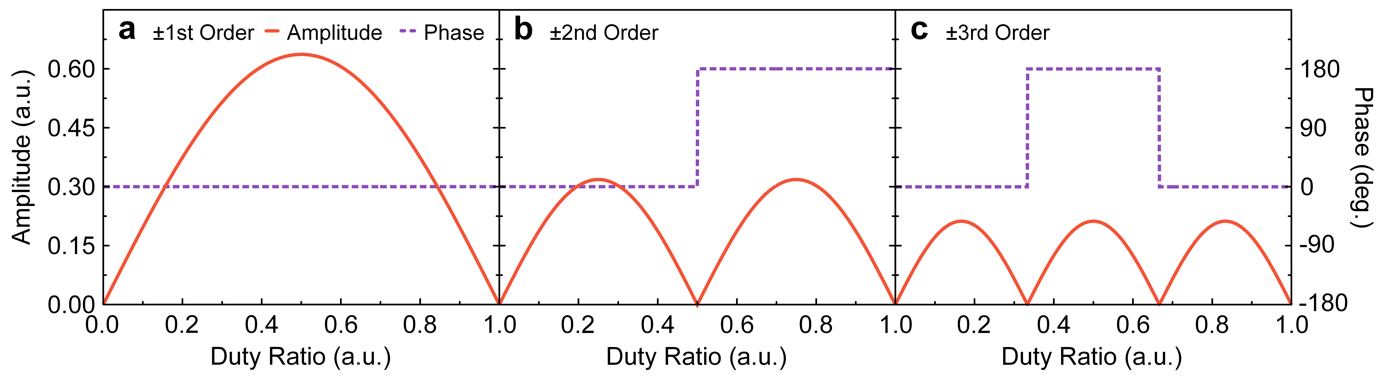


**Figure S1.** Calculated harmonic amplitudes (solid lines) and phases (dashed lines) versus the duty ratios at time delay *t*0=0. (a) The ±1st-order harmonics. (b) The ±2nd-order harmonics. (c) The ±3rd-order harmonics.

The above recipe endows us to tailor the harmonic amplitudes while keep the harmonic phases unchanged. Furthermore, we introduce the other parameter of the coding sequences that can modulate the harmonic phases while keep the harmonic amplitudes unaltered. In brief, since the amplitudes and phases of all harmonics are determined by the complex Fourier series coefficients of the periodic reflection coefficient , the time shift property is suitable herein to add an additional phase shift in the *k*th-order harmonic while keep its amplitude constant. The time shift property of the Fourier transform reads

, (S6)

in which *t*0 represents the time delay of periodic coding sequences. Hence we can recalculate the complex Fourier series coefficient of the *k*th-order harmonic as

, (S7)

which is actually the same as Eq. (1) in the main manuscript.

To better illustrate this principle, the dependence of harmonic phases with the time delay *t*0 is presented in Fig. S2, where the duty ratio is set as for the *k*th-order harmonic as well as ||=1, ||=1, =0°, and =180°. It is clear that the phase of the *k*th-order harmonic (solid line) is proportional to , in which . Moreover, when the time delay *t*0 varies from 0 to *T*0, the amplitudes of all harmonics remain unchanged only if the duty ratios are unaltered. Thus, when *t*0 is limited to , we can obtain a one-to-one correspondence between the *k*th-order harmonic phase and the time delay as well as an invariant harmonic amplitude.


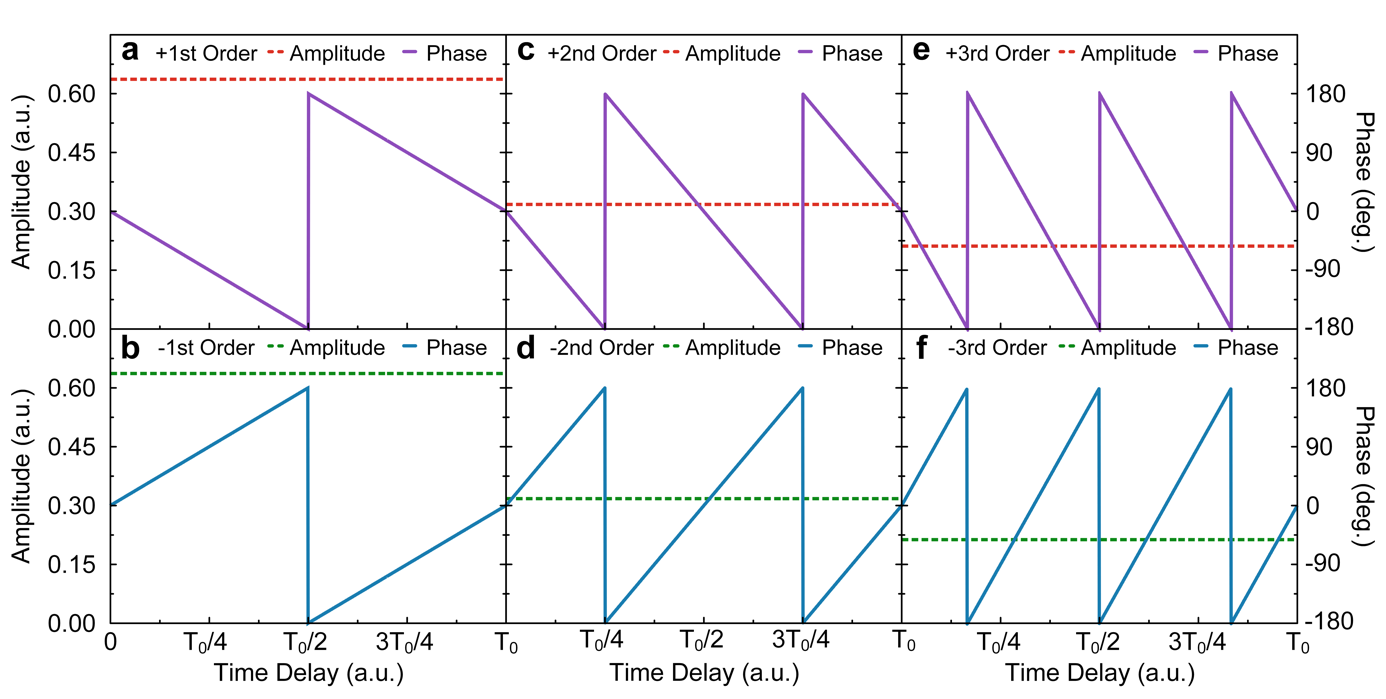


**Figure S2.** Calculated harmonic phases (solid line) and amplitudes (dashed line) versus the time delays at *M*=0.5 (the ±1st-order harmonics), *M*=0.25 (the ±2nd-order harmonics) and *M*=1/6 (the ±3rd-order harmonics). (a) The +1st-order harmonic. (b) The -1st-order harmonic. (c) The +2nd-order harmonic. (d) The -2nd-order harmonic. (e) The +3rd-order harmonic. (f) The -3rd-order harmonic.

To sum up, with aptly chosen ranges such as and for the +1st-order harmonic, the duty ratios and time delays of the digital coding sequences can accurately control the harmonic amplitudes and phases without any mutual influence.

**Supplementary Note 3: Analysis of harmonic manipulation in imperfect coding states of TDCM**

In the theoretical analysis in Supplementary Note 2, we assume two distinct coding states with uniform reflection amplitudes and opposite reflection phases (i.e. 0° and 180°) for simplicity. However, the coding states of TDCM in reality are not ideal due to designing and manufacturing deviations.

We reconsider the harmonic amplitude and phase distributions in Eq. (1) and (2). Let and , we have

. (S8)

In an ideal case of ||=1, ||=1, =0°, and =180°, is equal to , which has a maximal amplitude coefficient and zero initial phase. Figs S3a, c, and e show the calculated +1st-order harmonic amplitudes (solid line) and phases (dashed line) versus the duty ratios at the time delay *t*0=0, while Figs S3b, d, and f show the calculated +1st-order harmonic phases (solid line) and amplitudes (dashed line) versus the time delays at the duty ratio *M*=0.5 in imperfect coding states of TDCM. Generally speaking, the unideal coding states can be classified into three categories: unideal reflection amplitude, unideal reflection phase, and both. Firstly, when the reflection amplitude is unideal, as depicted in Fig. S3a and b, . Thus the efficiency in converting the fundamental wave to harmonics degrades as the harmonic phase distributions are unaffected. Secondly, when the reflection phase is unideal, as illustrated in Fig. S3c and d, not only the converting efficiency deteriorates but an extra initial phase is added to the harmonic phases. Finally, when both of the reflection amplitude and phase are unideal, as illustrated in Fig. S3e and f, the converting efficiency further deteriorates and the problem of extra initial phase exists at the same time.

From the above analysis, we find that since is a combination of amplitude coefficient and initial phase attached to all harmonics simultaneously, it has no influence in the relative dependence between the harmonic amplitudes and duty ratios, as well as between the harmonic phases and time delays. As a result, our mechanism renders TDCM to function as broadband beam shaper and wireless transmitter.


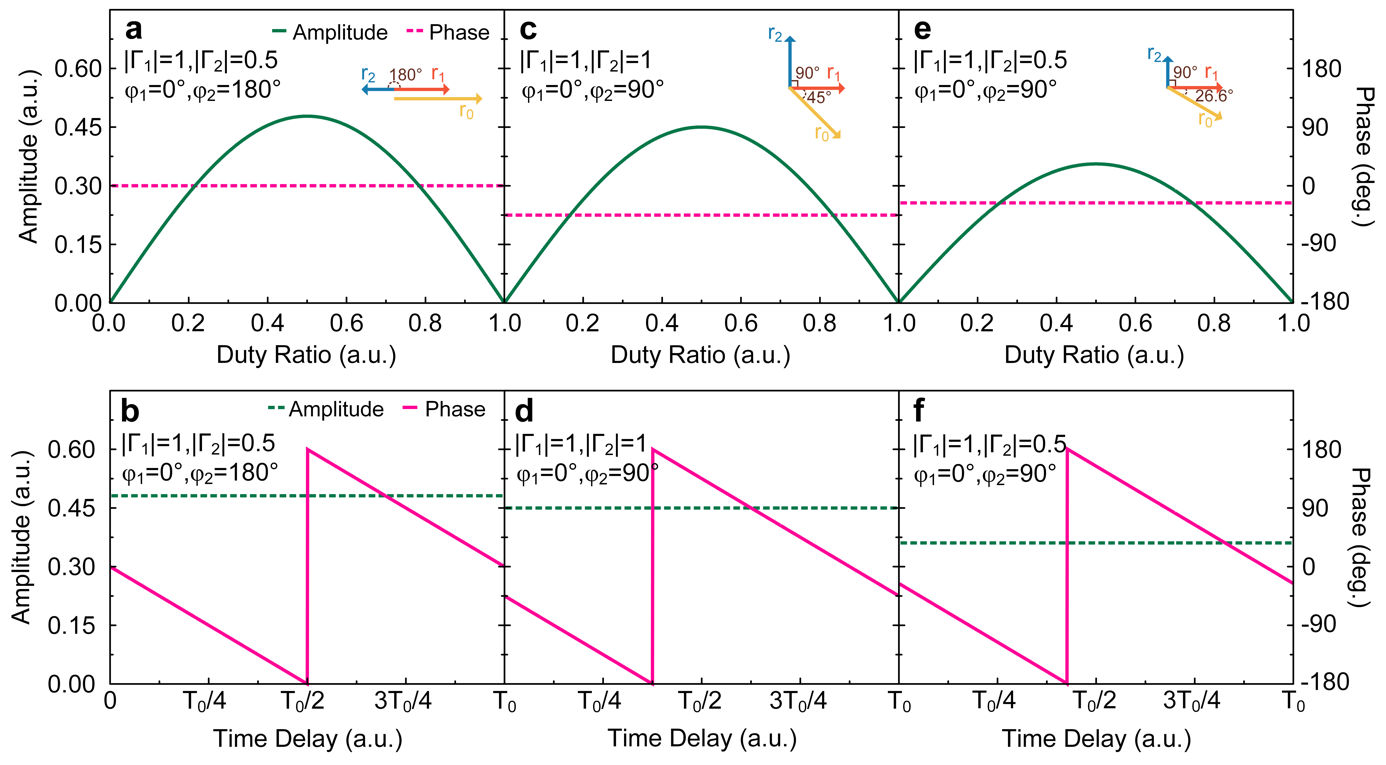


**Figure S3.** Calculated +1st-order harmonic amplitudes (solid line) and phases (dashed line) versus the duty ratio at the time delay *t*0=0 (a-e), as well as harmonic phases (solid line) and amplitudes (dashed line) versus the time delay at the duty ratio *M*=0.5 (b-f) in unideal coding states of TDCM. (a-b) ||=1, ||=0.5, =0°, and =180°. (c-d) ||=1, ||=1, =0°, and =90°. (e-f) ||=1, ||=0.5, =0°, and =90°.

**Supplementary Note 4: Experimental setup of harmonic beam steering**

As shown in the Experimental Verification part in the main text, the fabricated TDCM is composed of 56 columns of elements independently controllable via DIO signals. Note that merely 48 of the 56 columns are controlled in our experiment due to the constraints of DIO lines. The width of each element is 1.4 mm along the electric-field polarization direction. During the experiments, every 4 columns share the same control signal and form a super-column. As presented in Fig. S4, the experiments are set up in a microwave anechoic chamber to reduce interference. The Tx and Rx horn antenna which are respectively connected to the signal generator and spectrum analyzer, are put in the far-field region of the TDCM. In the meantime, 12 different coding sequences are simultaneously loaded to the super-columns via the DIO module. Finally, the measured directional diagrams are acquired by plotting received signal energies at different rotating angles.


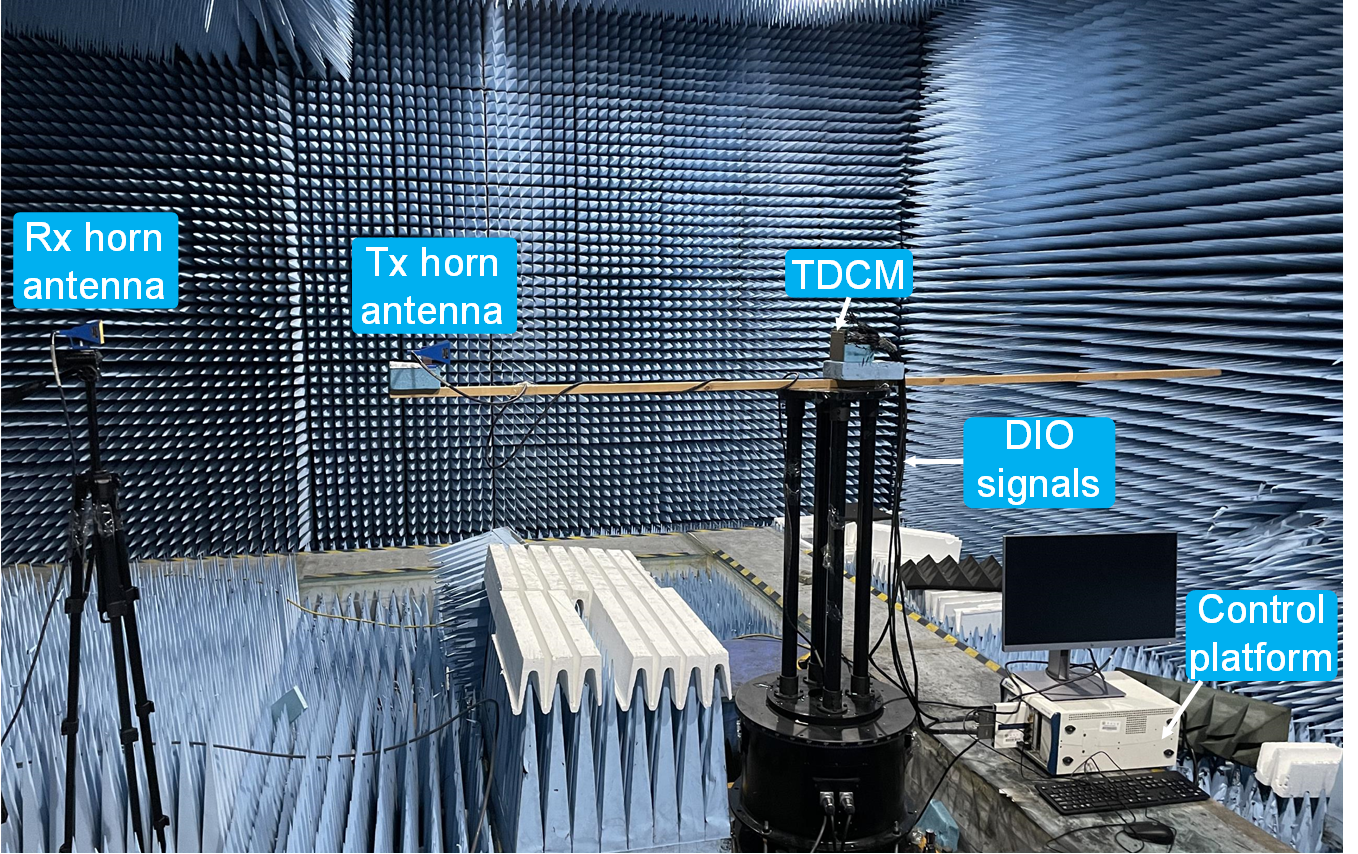


**Figure S4.** Photograph of the experimental setup. TDCM controlled by DIO signals from the control platform is placed in the far-field region of both Tx and Rx horn antennas.

**Supplementary Note 5: Impact of duty ratios *M* on harmonic beam gains**

Different duty ratios *M* are exerted at the coding sequences to tailor the gains of harmonic beams, in order to demonstrate the accuracy of harmonic amplitude controls. In Fig. S5, three phase distributions ‘000000000000’, ‘000066660000’, and ‘660066006600’ are selected in the experiment. *M*=0.5, 0.19, and 0.1 correspond to 0, -5, and -10 dB gains, which are respectively represented in red, blue, and green lines. The carrier wave frequency and modulating frequency are set as 27 GHz and 100 kHz, respectively. Meanwhile, the experiments are carried out at the +1st-order harmonic without losing generality. By comparing Fig. S5a with b, c with d, and e with f, the experimental gains of main lobes differ by 5 dB between different duty ratios *M*, aligning well with those of calculations, despite environmental interferences. As a result, the measurements solidly support our method over manipulating the harmonic amplitudes.


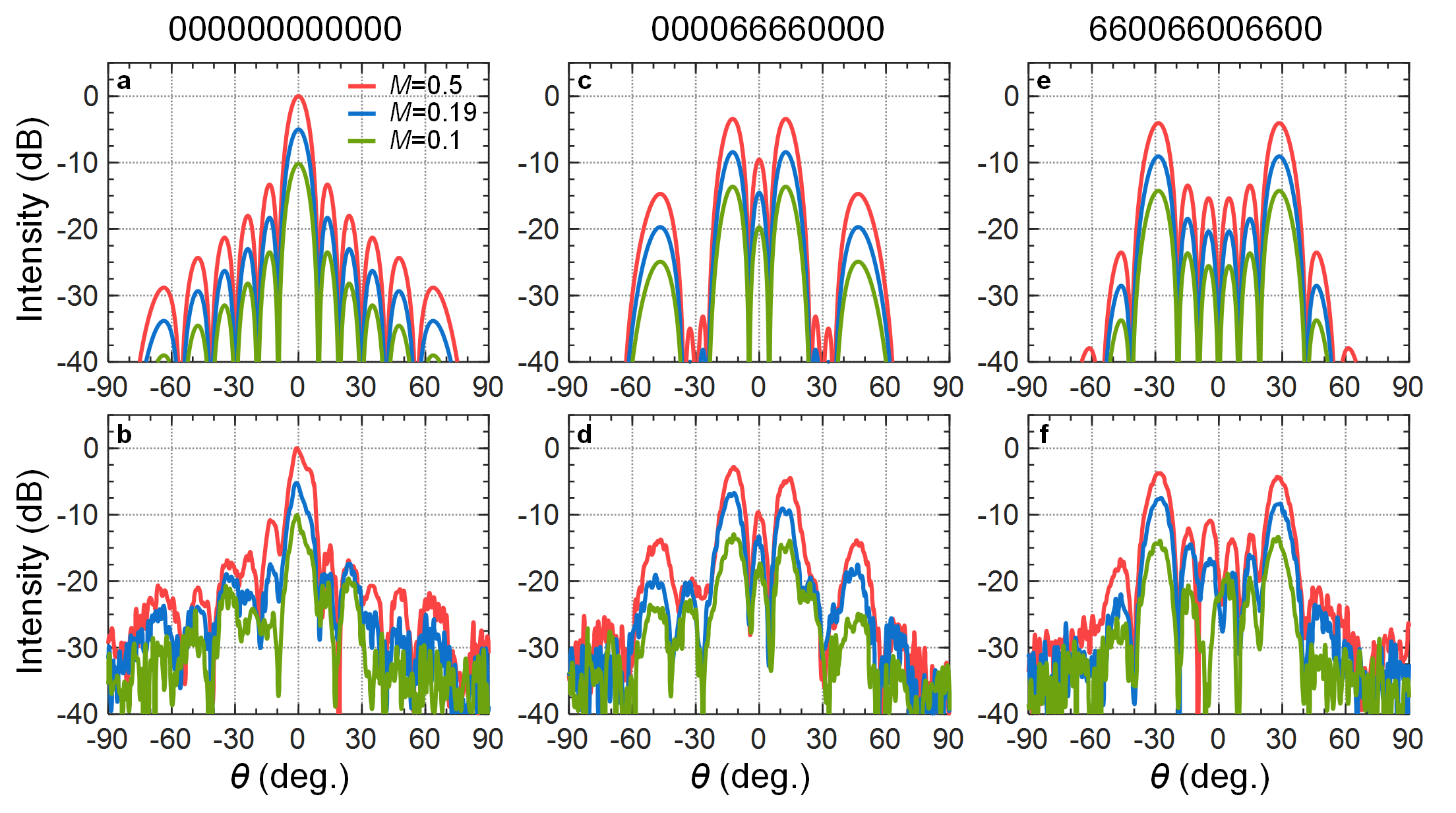


**Figure S5.** Calculated (a, c, e) and experimental (b, d, f) results for the +1st-order harmonics of TDCM at *f*c=27 GHz and *f*0=100 kHz. Three phase distributions listed at above are applied under different duty ratios, in which the red line stands for *M*=0.5, blue line for *M*=0.19, and green line for *M*=0.1, to demonstrate the accurate harmonic amplitude controls.

**Supplementary Note 6: Detailed deflection angles in Fig. 3**

**Table S1.** Simulated and measured deflection angles in Figs. 3a-d.

| Harmonic order | -3rd order | -1st order | +1st order | +3rd order |
| --- | --- | --- | --- | --- |
| Simulated angle (º) | 13.9 | 4.6 | -4.6 | -13.9 |
| Measured angle (º) | 14.8 | 4.1 | -3.9 | -14.7 |

**Table S2.** Simulated and measured deflection angles in Figs. 3e-h.

| Harmonic order | -3rd order | -1st order | +1st order | +3rd order |
| --- | --- | --- | --- | --- |
| Simulated angle (º) | -29.4 | 29.4 | -29.4 | 29.4 |
| Measured angle (º) | -29.1 | 29.9 | -29.6 | 30.7 |

**Table S3.** Simulated and measured deflection angles of state 2 at different frequencies in Figs. 3i-p.

| Frequency (GHz) | 22 | 25 | 30 | 33 |
| --- | --- | --- | --- | --- |
| Simulated angle (º) | -5.7 | -5.0 | -4.1 | -3.8 |
| Measured angle (º) | -5.4 | -4.1 | -3.8 | -4.0 |

**Table S4.** Simulated and measured deflection angles of state 3 at different frequencies in Figs. 3i-p.

| Frequency (GHz) | 22 | 25 | 30 | 33 |
| --- | --- | --- | --- | --- |
| Simulated angle (º) | -11.6 | -10.2 | -8.5 | -7.7 |
| Measured angle (º) | -11.8 | -10.5 | -7.5 | -7.9 |

**Table S5.** Simulated and measured deflection angles of state 4 at different frequencies in Figs. 3i-p.

| Frequency (GHz) | 22 | 25 | 30 | 33 |
| --- | --- | --- | --- | --- |
| Simulated angle (º) | -36.7 | -31.9 | -26.3 | -23.8 |
| Measured angle (º) | -36.1 | -32.9 | -26.4 | -24.2 |

**Supplementary Note 7: Comparison between +1st-order harmonic and higher-order harmonics in harmonic signal modulation**

In the main text, we remark that any *k*th-order harmonic can be utilized to realize signal modulation, but only +1st-order harmonic is actually adopted in experimental implementation. This is because the converting efficiency of +1st-order harmonic is much higher than other higher-order harmonics. The maximal amplitude of *k*th-order harmonic equals , which is inversely proportional to.

Here, an example is employed to exhibit the advantages of +1st-order harmonic, as shown in Fig. S6. +1st, +2nd, and +3rd-order harmonics are selected to implement QPSK modulation. Fig. S6a presents the coding sequences of three different harmonics to transmit four symbols of “00”, “10”, “11”, and “01”, where red and blue block represents two coding states of the TDCM. To obtain the respective duty ratio of 1/2, 1/4, and 1/6, the length of the coding sequences is 8, 16, and 24 for +1st, +2nd, and +3rd-order harmonics respectively. Hence the relative amplitudes of the constellation points in Fig. S6b are 1, 1/2, and 1/3 for +1st, +2nd, and +3rd-order harmonics, respectively. As a result, the +1st-order harmonic has the highest converting efficiency.

In conclusion, the +1st-order harmonic has the best performance in signal modulation compared to other higher-order harmonics.


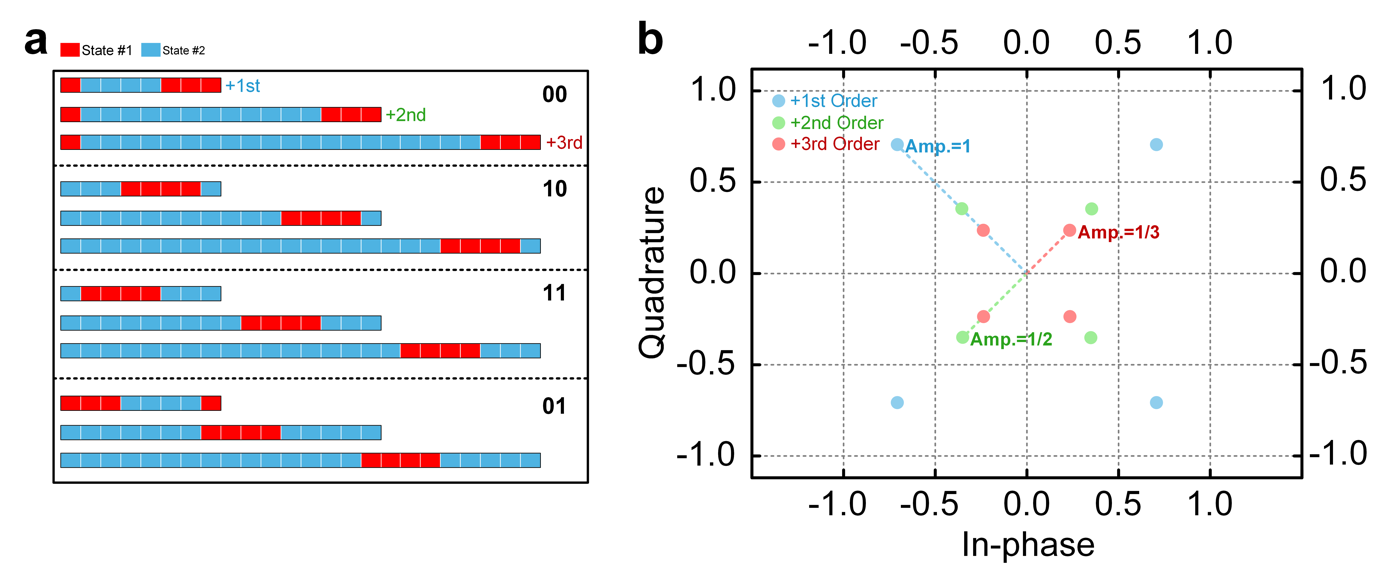


**Figure S6.** (a) Coding sequences of QPSK modulation for +1st-, +2nd-, and +3rd-order harmonic. (b) Constellation diagrams of QPSK modulation for +1st-, +2nd-, and +3rd-order harmonic.

**Supplementary Note 8: Mapping from transmission bits to digital coding sequences**

Based on the relationship between the constellation distributions and coding sequences, we obtain concrete mappings for the constant-envelope (BPSK, QPSK, and 8PSK) modulations, 16QAM, 64QAM, and 256QAM. The mappings for constant-envelope modulation and 16QAM are shown in Tabs S6-9. It is tedious to present 64QAM and 256QAM here. The readers can readily get them using the same method.

**Table S6.** Mapping relationship amongst transmission bits,, and for BPSK modulation.

|  |  |  |
| --- | --- | --- |
|  |  |  |
| Transmission bits | 0 | 1 |

**Table S7.** Mapping relationship amongst transmission bits,, and for QPSK modulation.

|  |  |  |  |  |
| --- | --- | --- | --- | --- |
|  |  |  |  |  |
| Transmission bits | 00 | 10 | 11 | 01 |

**Table S8.** Mapping relationship amongst transmission bits,, and for 8PSK modulation.

|  |  |  |  |  |  |  |  |  |
| --- | --- | --- | --- | --- | --- | --- | --- | --- |
|  |  |  |  |  |  |  |  |  |
| Transmission bits | 000 | 001 | 010 | 011 | 111 | 110 | 100 | 101 |

**Table S9.** Mapping relationship amongst transmission bits,, and for 16QAM.

|  |  |  |  |  |  |  |  |  |
| --- | --- | --- | --- | --- | --- | --- | --- | --- |
|  |  |  |  |  |  |  |  |  |
| Transmission bits | 0000 | 0001 | 0010 | 0011 | 0100 | 0101 | 0110 | 0111 |
|  |  |  |  |  |  |  |  |  |
|  |  |  |  |  |  |  |  |  |
| Transmission bits | 1000 | 1001 | 1010 | 1011 | 1100 | 1101 | 1110 | 1111 |

**Supplementary Note 9: Detailed structure of the devised 1-bit TDCM**

The detailed parameters of the meta-element are illustrated in Figs. S7a and b.

**
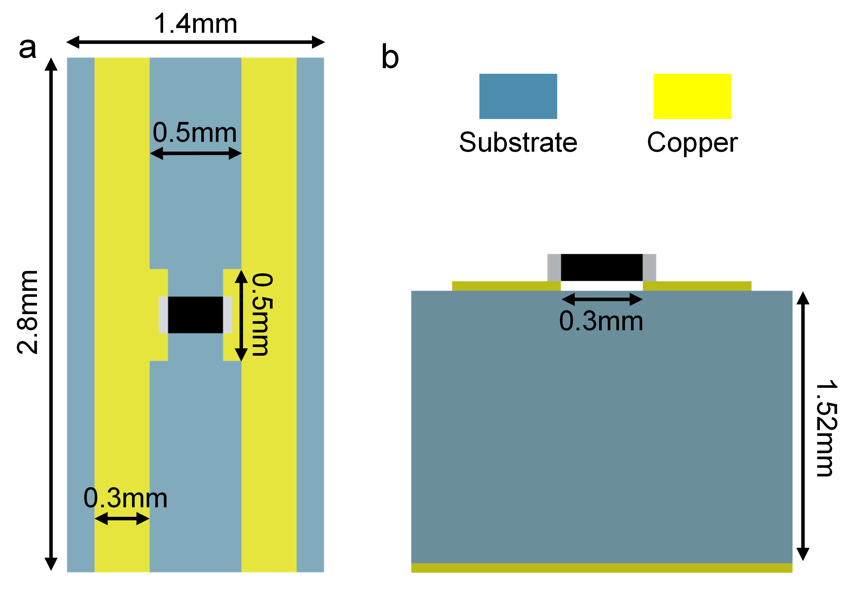
**

**Figure S7.** Detailed structure of the devised 1-bit TDCM. (a) Top view of the meta-atom. (b) Front view of the meta-atom.

**Supplementary Note 10: Measured harmonic amplitude and phase distributions**

To validate the harmonic-control theory presented in the main text, we conduct measurements of harmonic amplitudes and phases, when different coding sequences with varying duty ratios *M* and time delays *t*0 are loaded to TDCM under the illumination of carrier frequencies *f*c=27, 29, and 31.15 GHz, as shown in Fig. S8. We observe from the results that the carrier frequency *f*c has nearly no impact on the harmonic distributions except for the converting efficiency of the fundamental components, thus vividly verifying the broadband property of our method. We also note that the distributions of harmonic amplitudes are only dependent on the duty ratio *M*; while the harmonic phases are merely determined by the time delay *t*0. The major amplitude deviations between the measurements and calculations occur at the fundamental components, mainly when the coding states of TDCM are non-ideal. Moreover, the slight phase divergence primarily stemmed from the phase noise of the equipment. In short, despite of these measuring errors, the tested harmonic distributions strongly support the proposed broadband theory of harmonic manipulations.


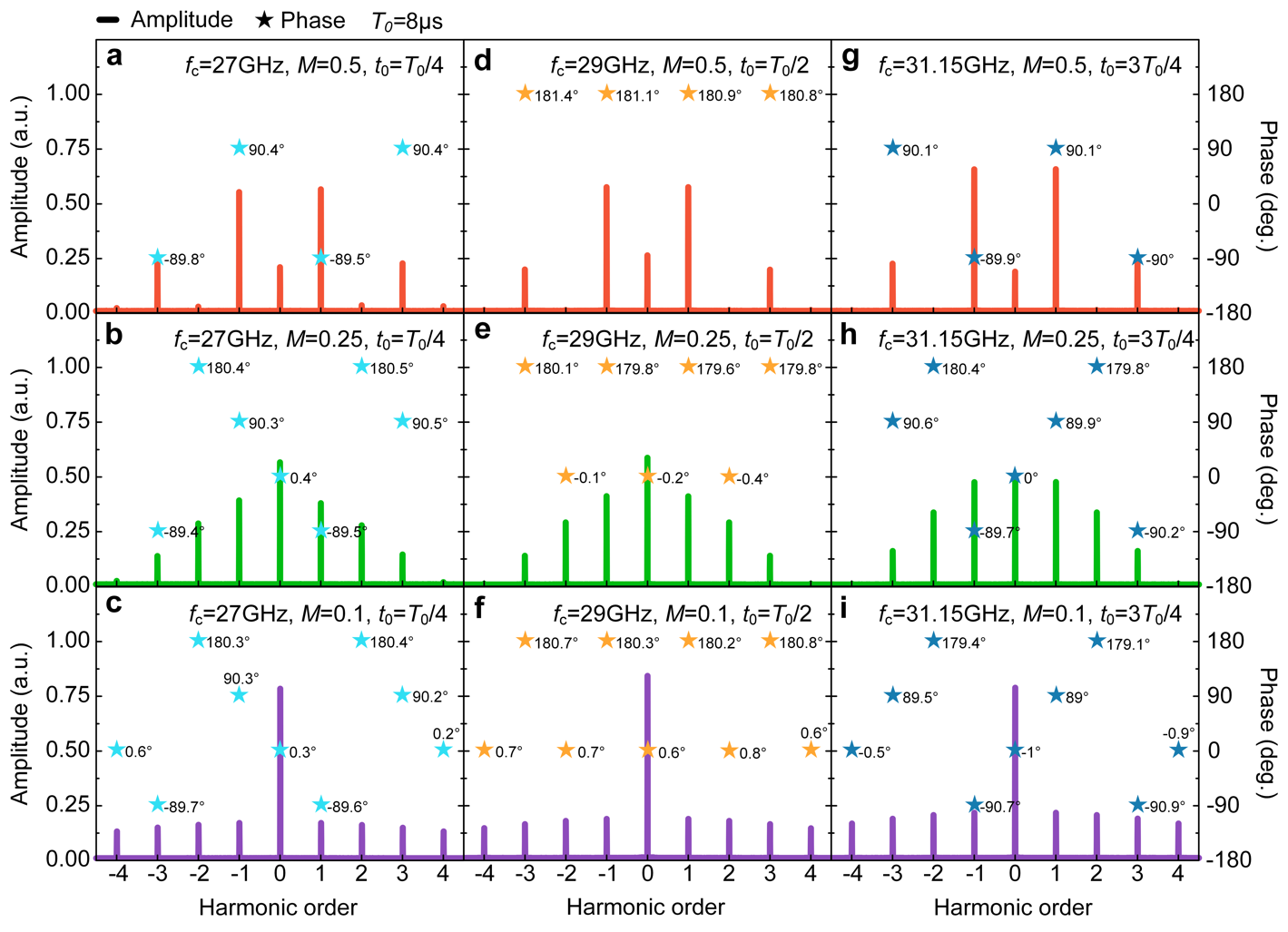


**Figure S8.** Measured harmonic amplitudes and phases under different carrier frequency *f*c, duty ratios *M*, and time delays *t*0 of the coding sequences at . Note that the phases are relative phases with regard to those of the same coding sequences without time delays. The same duty ratio (a, d, g), (b, e, h), and (c, f, i) are adopted in each row, while the same carrier frequency and time delays (a-c), and (d-f), as well as and (g-i) are employed in each column, respectively.

**Supplementary Note 11: Measured constellation diagrams of diverse modulation schemes with different modulation frequencies**

The measured constellations agree well with the standard constellations, as presented in Fig. S9. We clearly observe from Figs S9a-c that the quality of measured constellations degrade as the harmonic interval increases gradually, which can be ascribed to the phase noises in the RF module and distortion of biasing control voltages. Despite to these minute defects, an maximum communication rate of 37.5 Mbps is realized on the 8PSK modulation scheme, which is a great improvement compared with the previous works. In the meantime, due to the limited data rate of the DIO module (100 MS/s), the number of samples within a period is restricted (e.g. when the harmonic interval is set at 2.5 MHz, the number of samples within a period is 40 at most), thereby inducing quantization errors of the harmonic amplitudes and phases in high-order modulations. By comparing Fig. S9d with f and Fig. S9g with i, we can perceive that the precision of the constellation mapping degrades gradually when the harmonic interval increases. However, Fig. S9d and g indicate that as long as the desired harmonic amplitude and phase resolutions are satisfied, high-precision and high-order modulations could be accomplished using the proposed theory.


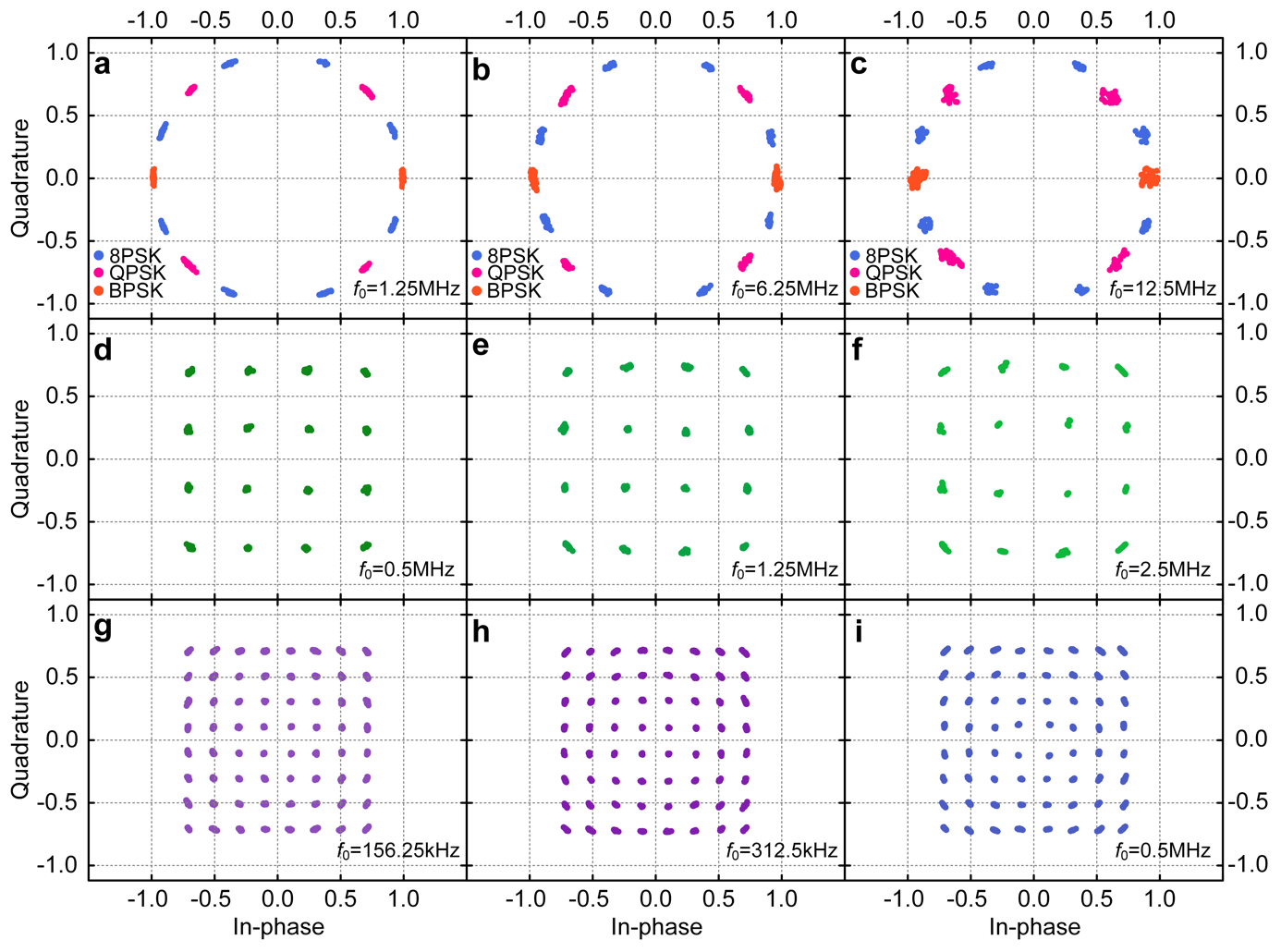


**Figure S9.** Measured constellation diagrams of diverse modulation schemes with different modulation frequencies at *fc*=27 GHz. (a-c) The constant envelope (BPSK, QPSK, and 8PSK) modulations with 1.25 MHz, 6.25 MHz, and 12.5 MHz frequency intervals, respectively. (d-f) 16QAM with 0.5 MHz, 1.25 MHz, and 2.5 MHz frequency intervals, respectively. (g-i) 64QAM with 156.25 kHz, 312.5 kHz, and 0.5 MHz frequency intervals, respectively. Owing to the extremely accurate control over harmonics, the communication system works well under different modulation schemes.

**Supplementary Video**

The process of transmitting images in the devised wireless communication system is truthfully recorded in the supplementary video, in which the 256QAM scheme with 1.25 Mbps data-transmission rate (frequency interval *f*0=156.25 kHz) is applied, and the carrier frequency *f*c is 27 GHz. Two images (the auditorium and library of Southeast University) are recurrently sent by the transmitter. Meanwhile, the receiver captures and deals with the signals, and then displays the real-time constellation diagrams and recovers the images in the screen. As is vividly demonstrated in the video, the received constellation diagrams and pictures remain stable amid a steady communication channel. Subsequently, when blockage occurred, the signal-noise-ratio (SNR) of the receiver diminishes, resulting in deterioration of the constellation diagrams and recovered images.

**References**

[1] Zhao J, Yang X and Dai JY *et al*. Programmable time-domain digital-coding metasurface for non-linear harmonic manipulation and new wireless communication systems. *Natl Sci Rev* 2019; **6**: 231-8.
